# Effect of a project achievement-oriented Quality Control Circle on traditional Chinese medicine nursing outcomes in elderly patients with distal radius fractures: A retrospective comparative study

**DOI:** 10.1097/MD.0000000000050905

**Published:** 2026-10-02

**Authors:** Na Cao, Ya-Mei Bai, Wen-Ting Wu, Fei-Yu Cheng, Ying Chen, Ming Zhou, Yang Jiang, Ting Tang, Han Sun

**Affiliations:** a School of Nursing, Nanjing University of Chinese Medicine, Nanjing, Jiangsu, China; b TCM Orthopedics and Traumatology Department, Changzhou Municipal Hospital of Traditional Chinese Medicine, Changzhou, Jiangsu, China; c Department of Nursing, Changzhou Municipal Hospital of Traditional Chinese Medicine, Changzhou, Jiangsu, China.

**Keywords:** comprehensive model, elderly distal radius fracture, Quality Control Circle, retrospective comparative study, traditional Chinese medicine nursing

## Abstract

This study aimed to evaluate the effectiveness of a project achievement-oriented Quality Control Circle (QCC) model in improving traditional Chinese medicine (TCM) nursing outcomes for elderly patients with conservatively treated distal radius fractures (DRFs). This retrospective comparative study included 112 elderly DRF patients admitted between June 2022 and December 2023. Based on the admission period, 56 patients (June 2022–March 2023) received routine care (control group), and 56 patients (March–December 2023) received a comprehensive TCM nursing model driven by a project-oriented QCC. Outcomes included complication rate, TCM intervention delivery rate, pain (Visual Analog Scale), swelling score, wrist function (Cooney score), and patient/staff satisfaction. Both groups received identical Menghe School bone-setting and small splint fixation. The intervention group additionally received a QCC-driven comprehensive TCM nursing model. The intervention group showed significantly lower total complication rates (3.6% vs 19.6%, relative risk = 0.18, *P* = .008), a higher TCM delivery rate (100% vs 64%, *P* < .05), greater reductions in pain and swelling, and higher Cooney scores (95 vs 75, *P* < .05) compared with the control group. All 9 quality indicators met or exceeded targets. Staff competency and team cohesion also improved. This retrospective study suggests that a project-oriented QCC model may enhance TCM nursing quality and short-term clinical outcomes in elderly DRF patients. However, given the study’s observational design and single-center setting, these preliminary findings require validation through multicenter prospective trials with validated instruments and long-term follow-up. Robustness analysis using a propensity score-matched concurrent control group yielded consistent findings, supporting the validity of the primary results.

## 1. Introduction

Distal radius fracture (DRF) refers to a fracture occurring within 3 cm of the articular surface of the distal radius. It is one of the most common fractures in individuals over 65 years of age, accounting for approximately 18% of all fractures, with an annual global incidence ranging from 0.04% to 1.10%.^[1,2]^ Surgical treatment for DRFs presents certain drawbacks. Traditional Chinese medicine (TCM) treatment, primarily based on closed reduction followed by plaster or splint fixation, can achieve favorable outcomes in about 70% of DRF cases.^[3,4]^ However, during treatment, complications such as osteofascial compartment syndrome, ischemic muscle contracture, splint loosening after swelling reduction, loss of reduction, and poor functional exercise may occur. Moreover, medical and nursing interventions are often fragmented. Therefore, constructing a comprehensive TCM nursing model program for elderly patients with DRFs is of paramount importance. Building on previous work, this study explores the establishment of a comprehensive TCM nursing management model for elderly DRF patients based on a project achievement-oriented Quality Control Circle (QCC), aiming to provide a reference for clinical nursing practice.^[5,6]^

Unlike traditional QCC activities that focus on process compliance, the project achievement-oriented QCC emphasizes measurable clinical endpoints and systematic integration of evidence-based TCM interventions into routine workflows. This shift from process-oriented to outcome-oriented quality improvement represents a novel strategy in TCM nursing management.^[7,8]^

## 2. Data and methods

### 2.1. General data

This retrospective comparative study was designed and reported in compliance with the Strengthening the Reporting of Observational Studies in Epidemiology guidelines for observational studies. This study employed a retrospective, non-randomized, historical control design. According to the order of admission, 56 patients admitted from June 2022 to March 2023 were assigned to the control group, and 56 patients admitted from March 2023 to December 2023 were assigned to the intervention group. The general data of the 2 groups are shown in Table 1. The allocation was based solely on admission time; no randomization was performed. No statistically significant differences were observed between the 2 groups in any baseline characteristics (*P* > .05, Table 1), indicating comparability at baseline. This study was approved by the Ethics Committee of Changzhou Hospital of Traditional Chinese Medicine (Approval No. 2022-LL-045 (S)). Written informed consent was obtained from all participants.

**Table 1:** Baseline demographic and clinical characteristics of elderly patients with distal radius fractures in the intervention and control groups.

Characteristic	Intervention (n = 56)	Control (n = 56)	Test statistic	DF	*P* value
Age, yr (mean ± SD)	71.2 ± 5.83	69.4 ± 3.67	*t* = 1.912	110	.059
Gender, n (%)			χ^2^ = 0.143	1	.705
Male	26 (46.4)	28 (50.0)			
Female	30 (53.6)	28 (50.0)			
Education level, n (%)			χ^2^ = 0.679	2	.712
≤Junior high school	15 (26.8)	13 (23.2)			
High school	24 (42.9)	23 (41.1)			
≥College	17 (30.4)	20 (35.7)			
Fracture laterality, n (%)			χ^2^ = 0.143	1	.705
Left	26 (46.4)	28 (50.0)			
Right	30 (53.6)	28 (50.0)			
AO/OTA classification, n (%)			χ^2^ = 0.144	1	.704
Type A	32 (57.1)	30 (53.6)			
Type B	24 (42.9)	26 (46.4)			

Continuous variables are presented as mean ± SD, and categorical variables are presented as n (%). No missing data were found in the baseline characteristics. All statistical tests were two-sided, and a *P* value < .05 was considered statistically significant.

χ^2^ = Pearson’s chi-square test, AO/OTA = AO Foundation/Orthopaedic Trauma Association, DF = degrees of freedom, SD = standard deviation, *t* = independent-samples *t* test.

#### 2.1.1. Inclusion criteria

1. Age ≥ 65 years.
2. Clear diagnosis of DRF by a physician.
3. Planned conservative treatment.
4. Good cognitive status and ability to cooperate with treatment.

#### 2.1.2. Exclusion criteria

1. Severe cardiopulmonary or cerebral dysfunction.
2. Presence of other fractures or open fractures.
3. Co-existing infections.
4. Receiving other concurrent treatments.
5. Incomplete data or uncooperative with the study.^[9]^

### 2.2. Outcome measures

#### 2.2.1. Primary outcomes

Overall complication rate (malunion, joint stiffness, nerve injury, infection); TCM intervention delivery rate.

#### 2.2.2. Secondary outcomes

Pain intensity: Visual Analog Scale (VAS, 0–10); limb swelling: 4-point ordinal scale (1 = none/mild, 4 = severe), developed by the department, with inter-rater reliability κ = 0.82; wrist function: Cooney wrist function score (0–100, higher = better); TCM syndrome efficacy score (0–100); patient satisfaction with TCM treatment; staff recognition of the model.

These measures were assessed independently by 2 nurses to minimize bias. Limb swelling was assessed using a 4-point ordinal scale developed by our department. Although inter-rater reliability was examined (Cohen’s κ = 0.82), the scale has not been formally validated. Results should therefore be interpreted with caution.

All outcomes were assessed at baseline and at 8 weeks after treatment initiation.

### 2.3. Methods

#### 2.3.1. Control group (routine treatment and nursing)

The control group received the standard orthopedic treatment protocol for conservatively managed DRF, which included diagnosis of a DRF suitable for conservative treatment after outpatient/emergency examination; manual reduction performed by orthopedists using the 12 bone-setting techniques (Shierzhong Zhenggu Shoufa; a set of manual reduction maneuvers) of the Menghe School (Menghe Xuepai); see Appendix A, Supplemental Digital Content 1, for a glossary of TCM terms; small splint fixation after reduction; and patient health education and functional guidance. TCM external therapies (e.g., herbal ointments or oils) were not systematically applied in the control group and were used only at the treating physician’s discretion, without standardized protocols or nursing quality monitoring^[10]^ (see Table 2).

**Table 2:** Intervention comparison between study groups.

Intervention component	Control group	Intervention group
Basic orthopedic treatment	Menghe School bone-setting + small splint fixation	Menghe School bone-setting + small splint fixation
Nursing workflow	Standard ward pathway without integrated imaging	One-stop closed-loop service from admission to follow-up
Case management	No dedicated case manager	Full-time case manager + WeChat follow-up
Health education	Oral guidance + paper leaflets	Standardized video training + scenario simulation
Early TCM therapy (0–2 wk)	Non-standardized, physician-discretion only	Standardized Heiyu Ointment topical application
Middle TCM therapy (2–4 wk)	Non-standardized, irregular use	Standardized Shangjin Zhenggu Oil + functional training
Late TCM therapy (4–8 wk)	Non-standardized, rarely used	Staged Roujin Yangjin Zhenggu Ointment application
Quality management	Monthly routine QC inspection only	Full-process QCC monitoring + performance linkage

All basic treatments were identical between groups; TCM therapies were standardized in the intervention group per protocol.

QC = quality control, QCC = Quality Control Circle, TCM = traditional Chinese medicine.

#### 2.3.2. Intervention group (comprehensive TCM nursing model based on QCC)

The intervention group received the same orthopedic treatment protocol as the control group (Menghe School bone-setting and small splint fixation). In addition, a comprehensive TCM nursing model was implemented using the project achievement-oriented QCC methodology. This model established a one-stop medical service process of “admission–imaging–reduction–imaging” and utilized the QCC as a quality management tool throughout the entire treatment process, systematically controlling outcome indicators. Key differences from the control group included process optimization (e.g., a dedicated bone-setting imaging room and bed for immediate feedback); standardized, staged application of specific TCM preparations (Heiyu Ointment, Tendon Injury Bone-setting Oil, Roujin/Zhenggu Ointments); and the structured QCC methodology to drive these changes.

##### 2.3.2.1. Overview of the QCC methodology

This study employed the “project achievement-oriented” QC-Story approach, a variant of the standard QCC methodology specifically designed for creating new systems or achieving ambitious targets (as opposed to problem-solving). The complete QCC implementation followed the ten-step QC-Story framework (see Fig. 1):

**Figure 1.**
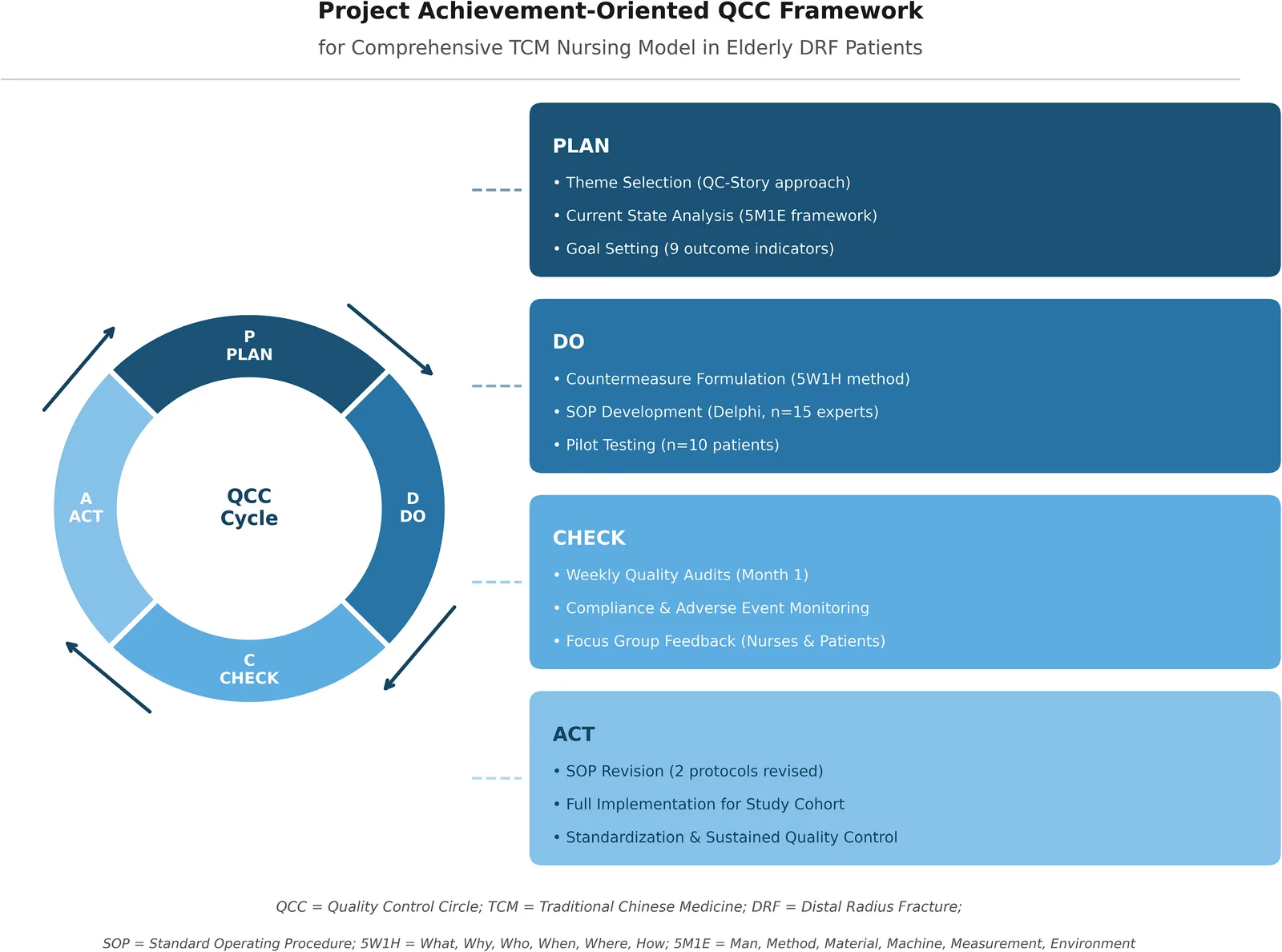
Flowchart of the comprehensive TCM nursing model implementation based on a project achievement-oriented QCC for elderly DRF patients. The flowchart includes 5 systematic phases: QCC team organization, theme selection, current state analysis and key issue identification, goal setting, and countermeasure formulation and implementation. DRF = distal radius fracture, QCC = Quality Control Circle, TCM = traditional Chinese medicine.

**Step 1** – Theme selection: Through structured brainstorming sessions, 4 candidate themes were proposed by the QCC team. Each theme was scored using a weighted matrix (policy alignment 30%, importance 25%, urgency 25%, circle capability 20%; maximum score 210 points). The selected theme, “Construction and Application of a Comprehensive TCM Nursing Model for Elderly DRF Patients,” scored 186 points, exceeding the threshold of 168 points, and was designated as a project achievement-oriented QCC activity.
**Step 2** – Activity planning: A Gantt chart was developed covering the entire QCC cycle (March 2023–December 2023). The plan allocated 2 weeks for team formation and training, 4 weeks for current state analysis, 2 weeks for goal setting, 6 weeks for countermeasure development, 12 weeks for implementation, and 8 weeks for effect confirmation and standardization.
**Step 3** – Current state analysis: A self-designed checklist was used to investigate collaborative management across Orthopedics, Pharmacy, and Publicity departments. Data were analyzed from 5 perspectives: personnel, methods, materials, information, and systems. Key findings were visualized using fishbone (Ishikawa) diagrams and Pareto charts. Five major issues were identified: absence of a dedicated TCM characteristic nursing team; lack of standardized comprehensive TCM nursing processes; insufficient hardware facilities and inconsistent bone-setting techniques; nonuniform health education materials; and limited information dissemination channels.
**Step 4** – Goal setting: Based on literature review, departmental historical data (2022 baseline), and QCC team capability assessment, 9 SMART (Specific, Measurable, Achievable, Relevant, Time-bound) outcome indicators were established with target values (Table 3).
**Step 5** – Analysis and key issue identification: All identified issues were scored based on feasibility (40%), economy (30%), and circle capability (30%). Five key improvement points exceeding the threshold of 168/210 were selected: improving medical staff cognitive level; constructing model guarantee schemes; improving total model compliance rate; enhancing TCM participation throughout the process; and increasing medical-nursing-patient communication channels.
**Step 6** – Countermeasure formulation: Using the 5W1H (What, Why, Where, When, Who, How) method, improvement countermeasures were formulated for each key point. Each countermeasure was scored on effectiveness (40%), feasibility (30%), and cost (30%). The optimal countermeasures were integrated into 3 policy groups (Table 4).
**Step 7** – Countermeasure implementation: The 3 policy groups were implemented sequentially over 12 weeks (June–August 2023). Weekly team meetings monitored progress using PDCA (Plan-Do-Check-Act) cycles. Implementation fidelity was assessed through direct observation and chart audits.
**Step 8** – Effect confirmation: Both tangible outcomes (9 clinical indicators) and intangible outcomes (team competency scores) were measured at 3 months post-implementation (November–December 2023). Comparison with baseline data and target values was performed (Table 9).
**Step 9** – Standardization: Four standardized operation manuals were developed and submitted for hospital review: Operation Procedure for Small Splint Fixation; Operation Procedure for External Application of TCM; Clinical Nursing Pathway for Elderly DRFs; and Evaluation Standards for Specialist Nursing Quality. These were incorporated into departmental routine assessment.
**Step 10** – Review and future planning: The QCC activity was presented at the 2023 Hospital QCC Competition (first prize) and the Jiangsu Provincial Hospital Association QCC Competition (second prize). Remaining issues (e.g., long-term follow-up system) were identified for subsequent QCC cycles.

**Table 3:** QCC preset indicators and target values.

Indicator category	Indicator name	Baseline	Target value
TCM-related indicators	Clinical implementation rate of TCM interventions	65.0%	100.0%
	TCM syndrome efficacy score	63	95
Orthopedic clinical indicators	Limb swelling score (higher = more severe)	3	1
	VAS pain score (0–10)	6	2
	DRF clinical healing rate	60.0%	90.0%
	Cooney wrist function score (0–100)	75	95
	DRF complication incidence rate	25.0%	3.0%
Patient and staff satisfaction indicators	Patient satisfaction rate with TCM treatment	81.0%	98.0%
	Medical staff recognition rate of the TCM nursing model	70.0%	99.0%

Baseline values represent routine nursing outcomes before QCC implementation (June 2022–March 2023).

Interpretation of scoring scales: higher limb swelling score indicates more severe edema; higher VAS pain score indicates greater pain intensity; higher Cooney wrist function score represents better wrist functional recovery.

All indicators were established following the SMART principle for QCC quality improvement monitoring.

DRF = distal radius fracture, QCC = Quality Control Circle, SMART = Specific, Measurable, Achievable, Relevant, Time-bound, TCM = traditional Chinese medicine, VAS = Visual Analog Scale.

**Table 4:** Formulation and weighted scoring of improvement countermeasures.

Core improvement dimension	Specific countermeasure	Weighted total score	Policy grouping
Improve medical staff’s cognitive level of the model	Establish integrated medical-nursing management team	124	I
	Build standardized TCM training knowledge base	128	
	Develop diversified offline + online training and assessment system	132	I
Construct model guarantee scheme	Formulate whole-process standardized diagnosis and treatment specifications	136	
	Clarify standardized operation workflow for orthopedic nursing	132	
	Set up full-time QC team and refine TCM nursing implementation plan	122	
	Unify standardized health education publicity materials	128	
Improve total model compliance rate	Standardized Gartland fracture grading management	140	
	Regular DASH upper limb function assessment management	120	
Improve TCM participation rate in the model	Construct independent dedicated bone-setting operation room	128	II
	Configure special bone-setting nursing beds	136	
	Stage-based external application of Ma’s Five-color Ointment according to fracture symptoms	124	
	Standardized external use of Tendon Injury Bone-setting Oil	136	
	Staged application of Roujin and Yangjin Zhenggu Ointment	136	
Diversify medical-nursing-patient communication channels	Establish WeChat continuous health management groups for DRF patients	124	III
	Develop multi-channel offline + online health publicity methods	136	

Weighting rule: total score = effectiveness (40%) + feasibility (30%) + cost (30%); full score = 210 points. Countermeasures with a total score ≥168 points were retained for final implementation. Policy grouping definition: Group I = core system and management countermeasures; Group II = TCM characteristic intervention countermeasures; Group III = patient-centered communication and health management countermeasures. All countermeasures were predefined in the study protocol, independently scored by 3 core members of the QCC team, and approved by the institutional nursing committee.

DASH = Disabilities of the Arm, Shoulder and Hand Outcome Measure, DRF = distal radius fracture, QC = quality control, QCC = Quality Control Circle, TCM = traditional Chinese medicine.

**Table 9:** Comparison of all preset outcome indicators before and after QCC implementation.

Indicator	Pre-QCC baseline	Post-QCC actual result	Improvement (Δ)	Preset target value
VAS pain score (0–10)	6	2	↓ 4.0	2.0
Limb swelling score (1–4)	3	1	↓ 2.0	1.0
DRF clinical healing rate (%)	65.0	90.0	↑ 25.0	90.0
Cooney wrist function score (0–100)	75	95	↑ 20.0	95.0
DRF total complication incidence (%)	25.0	3.0	↓ 22.0	3.0
Clinical implementation rate of TCM interventions (%)	65.0	100.0	↑ 35.0	100.0
TCM syndrome efficacy score (0–100)	63	95	↑ 32.0	95.0
Patient satisfaction rate with TCM treatment (%)	81.0	98.0	↑ 17.0	98.0
Medical staff recognition rate of TCM nursing model (%)	70.0	99.0	↑ 29.0	99.0

↓ indicates improvement with a reduced value; ↑ indicates improvement with an elevated value. All indicators achieved or exceeded the preset target values post-intervention.

DRF = distal radius fracture, QCC = Quality Control Circle, TCM = traditional Chinese medicine, VAS = Visual Analog Scale.

##### 2.3.2.2. Establishment of the QCC team

This activity was patient-centered and based on medical-nursing collaboration and multidisciplinary team cooperation. The head nurse of the orthopedics department served as the circle leader, responsible for managing and organizing QCC activities. The director of the Provincial Department of Orthopedics and Traumatology TCM Nursing served as the advisor, responsible for coordination and supervision. The circle was named the “Bone-setting Circle,” signifying the use of Menghe School bone-setting manipulation and characteristic TCM nursing techniques to treat elderly DRF patients.

##### 2.3.2.3. Theme selection

Through brainstorming, 4 improvement themes were proposed and scored based on superior policy alignment, importance, urgency, and circle capability. The final theme selected was “Construction and Application of a Comprehensive TCM Nursing Model for Elderly Distal Radius Fractures,” designated as a project achievement-oriented QCC using the QC-Story approach.

##### 2.3.2.4. Current state analysis and key issue identification

Understanding the Status Quo: A self-designed checklist was used to investigate the current state of collaborative management for elderly DRFs across the Orthopedics, Pharmacy, and Publicity departments from the perspectives of personnel, methods, materials, information, and systems. Identified issues included lack of a dedicated TCM characteristic team; absence of standardized comprehensive TCM nursing processes; insufficient hardware facilities and inconsistent bone-setting techniques; lack of uniform health education materials; and limited information dissemination channels. Determining key improvement points: All identified issues were scored based on feasibility, economy, and circle capability (full score: 210; threshold: 168). Five key points were integrated and finalized: improving medical staff’s cognitive level of the model, constructing model guarantee schemes, improving total model compliance rate, enhancing the participation rate of TCM throughout the process, and increasing medical-nursing-patient information dissemination channels.

##### 2.3.2.5. Goal setting

Based on the survey and literature review, 9 outcome indicators with target values were established (Table 3).

##### 2.3.2.6. Formulation and selection of countermeasures

Using the 5W1H method, improvement countermeasures were formulated for the 5 key points and scored. The optimal countermeasures were integrated into 3 policy groups (Table 4): construction of an integrated management model; achieving full coverage of the TCM diagnosis and treatment model; and improving education content and methods, and optimizing communication channels.

##### 2.3.2.7. Implementation of optimal countermeasures

1. Construction of an integrated TCM management model with a clear division of responsibilities.
2. Comprehensive coverage of the TCM nursing model. Prior to the activity, care for DRF patients was fragmented. Referring to relevant guidelines, the nursing pathway for elderly DRFs was refined, including optimizing the entire process from admission to discharge and follow-up via information systems and WeChat groups managed by case managers; standardizing operating procedures for small splint fixation, external application of Ma’s Five-color Ointment and Tendon Injury Bone-setting Oil; and formulating a TCM nursing plan for elderly DRFs. Nursing quality evaluation standards were also developed and incorporated into departmental assessment.
3. Enriching health education content using digital tools. Previously, medical staff’s knowledge and educational methods were limited. Training frequency and themes were established through the online “512 Jingyi system” (an internal online training platform) and offline methods, combining lectures and scenario-based exercises. Training covered DRF basics, bone-setting demonstrations, and TCM operation assessment. Educational videos on orthopedic science and upper limb functional exercises were produced to diversify health education formats.^[11–14]^

#### 2.3.3. Robustness analysis: propensity score-matched concurrent control

To address the potential temporal confounding inherent in the historical control design (control group: June 2022–March 2023; intervention group: March–December 2023), we performed a supplementary robustness analysis using a propensity score-matched concurrent control group.

##### 2.3.3.1. Source and identification of concurrent controls

The QCC-based comprehensive TCM nursing model was implemented in a phased, ward-by-ward rollout starting in March 2023, rather than as a hospital-wide switch. During the intervention period (March–December 2023), patients admitted to the intervention wards (Ward A and Ward B) received the QCC-driven comprehensive TCM nursing model, while patients admitted to nonintervention wards (Ward C and Ward D) during the same period received routine care only. This ward-based allocation was determined by the hospital’s staged implementation schedule and staff training plans, rather than by patient-level characteristics, thereby reducing the risk of selection bias.

##### 2.3.3.2. Screening and matching process

From the hospital’s electronic medical record database, we identified all elderly DRF patients who met the same inclusion and exclusion criteria and were admitted during the intervention period (March–December 2023) but did not receive the QCC-based comprehensive TCM nursing model (i.e., received routine care only). Using 1 ∶ 1 nearest-neighbor propensity score matching without replacement, we matched each intervention group patient to a concurrent control patient based on the following baseline covariates: age, sex, body mass index, fracture classification (AO Foundation/Orthopaedic Trauma Association type), comorbidities (hypertension, diabetes, osteoporosis), and baseline VAS pain score. Propensity scores were estimated using a multivariable logistic regression model with group assignment (intervention vs concurrent control) as the dependent variable. A caliper width of 0.2 times the standard deviation of the logit of the propensity score was applied to ensure close matching. Balance of covariates between the matched groups was assessed using standardized mean differences (SMDs), with SMD < 0.1 indicating acceptable balance. A flowchart illustrating the participant selection process for the propensity score matching analysis is provided in Figure 2. After matching, all outcome measures (complication rate, TCM delivery rate, VAS pain score, swelling score, Cooney wrist function score, and patient satisfaction) were compared between the intervention group and the propensity score-matched concurrent control group using the same statistical methods as in the primary analysis.

**Figure 2.**
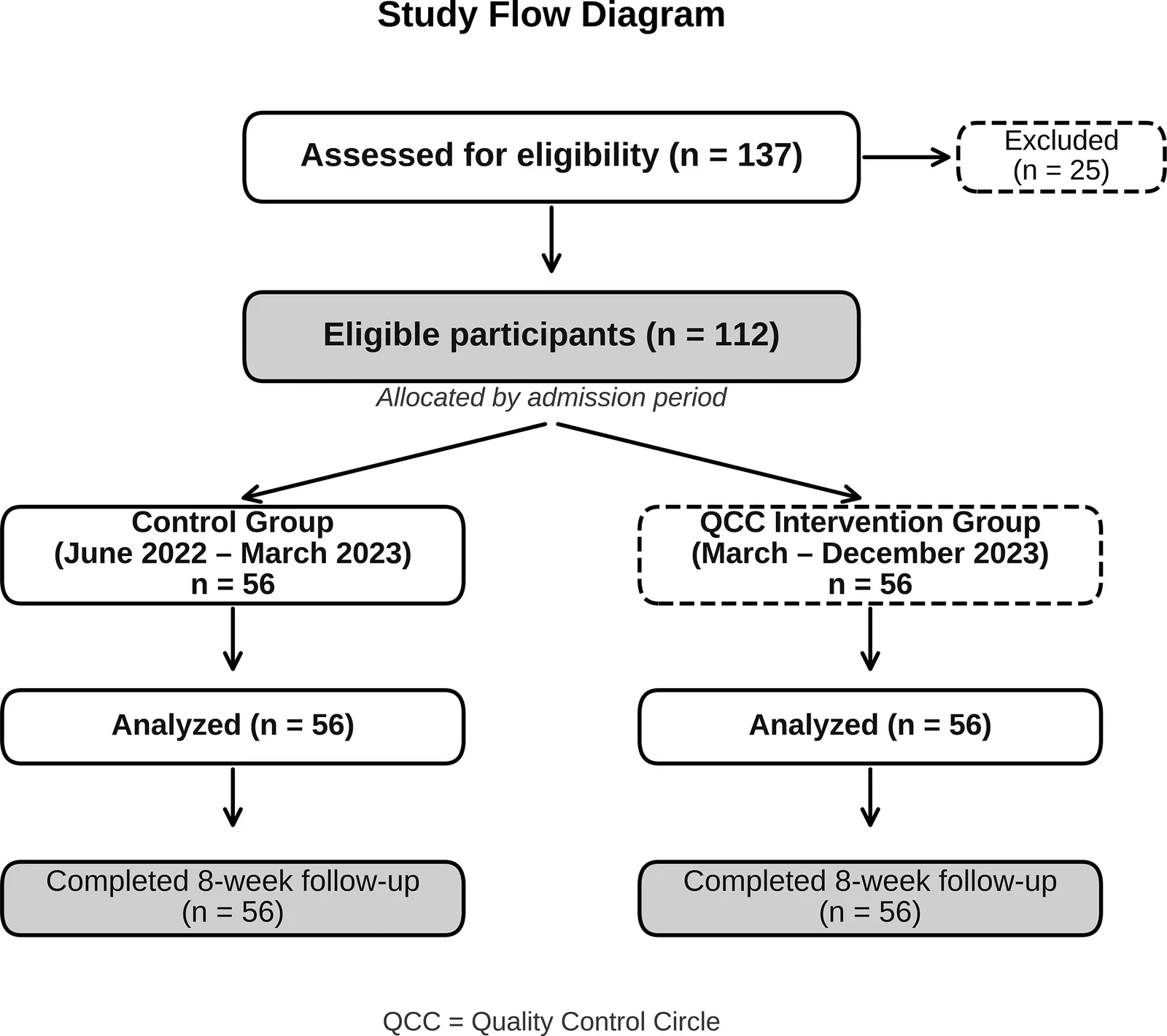
Study flow diagram of elderly patients receiving conservative treatment for DRF. A total of 137 elderly DRF patients were screened; 25 were excluded, leaving 112 eligible participants assigned to 2 cohorts by admission period: the historical control group (enrollment: June 2022–March 2023, n = 56) and the QCC intervention group (enrollment: March–December 2023, n = 56). All 112 participants completed the scheduled 8-week follow-up without loss to follow-up. DRF = distal radius fracture, QCC = Quality Control Circle.

### 2.4. Statistical methods

Statistical analyses were performed using SPSS version 26.0 (IBM Corp.). Categorical variables were compared using the chi-square test or Fisher’s exact test, and continuous variables were analyzed using the independent-samples *t* test or Mann–Whitney *U* test, as appropriate. Relative risks (RRs) with 95% confidence intervals (CIs) were calculated for complication rates.

Given the exploratory nature of this quality improvement study and the use of predefined outcome indicators aligned with QCC methodology, no formal adjustment for multiple comparisons was performed. However, to mitigate the risk of type I error, we adopted a hierarchical testing approach: the 2 primary outcomes (complication rate and TCM delivery rate) were tested at α = 0.05; secondary outcomes were considered supportive and interpreted alongside effect sizes and 95% CIs rather than relying solely on *P* values. Readers should interpret these findings as hypothesis-generating rather than confirmatory.^[14]^ Additionally, given the exploratory nature and predefined QCC indicator framework, we prioritized clinical relevance and effect magnitude over strict type I error control for secondary outcomes.

The required sample size was determined a priori based on the primary outcome of overall complication rate. Referring to our departmental historical data (control group complication rate: 19.6%) and clinically expected reduction with the intervention (target complication rate: 3.6%), we calculated the sample size using a two-sided chi-square test for 2 independent proportions. With a significance level (α) of 0.05 and a statistical power (1 − β) of 0.80, the minimum required sample size was estimated to be 46 patients per group. Considering a potential 10% dropout or protocol deviation rate, we planned to enroll at least 52 patients per group. Ultimately, 56 patients per group were included in the analysis, which provided sufficient statistical power to detect the prespecified difference in the primary outcome. Sample size calculation was performed using PASS 2021 software (NCSS, LLC, Kaysville).

No missing data were present for any of the baseline or outcome variables in either group; therefore, no imputation methods were applied.

## 3. Results

### 3.1. Comparison of clinical efficacy between the 2 groups

All 112 patients completed the 8-week follow-up, with no losses or missing data for the primary outcomes. The mean follow-up duration was 8.0 ± 0.5 weeks. The total effective rate was observed to be significantly higher in the intervention group than in the control group (94.64% vs 80.36%, *P* = .008; Table 5). The total incidence of complications was 3.6% (2/56) in the intervention group, significantly lower than the 19.6% (11/56) in the control group (RR = 0.18, 95% CI: 0.04–0.78, *P* = .008; Table 5).

**Table 5:** Comparison of clinical efficacy and complication rates between the intervention and control groups in elderly patients with distal radius fractures.

Group	n	Total effective cases (effective rate)	Total complication rate	Complications, n (%)			
				Malunion	Joint stiffness	Nerve injury	Infection
Intervention	56	53 (94.64%)	3.6%	1 (1.8%)	1 (1.8%)	0 (0.0%)	0 (0.0%)
Control	56	45 (80.36%)	19.6%	4 (7.1%)	5 (8.9%)	1 (1.8%)	1 (1.8%)
χ^2^ value	–	7.049	7.049	–	–	–	–
*P* value	–	.008	.008	–	–	–	–

Categorical variables were compared using Pearson’s chi-square test. A two-sided *P* value < .05 was considered statistically significant.

Effective rate was defined as the proportion of patients with excellent or good clinical efficacy after treatment, assessed by the co-chief physicians of orthopedics.

Complications were diagnosed and confirmed by imaging and clinical examination, independently evaluated by 2 physicians blinded to the group allocation.

All statistical analyses were performed using SPSS 26.0 statistical software.

### 3.2. Comparison of satisfaction-related indicators between the 2 groups

The total patient satisfaction with TCM treatment and total medical staff recognition of the model were both higher in the intervention group compared with the control group (*P* < .05; see Table 6).

**Table 6:** Comparison of patient satisfaction and medical staff recognition between intervention and control groups for elderly patients with distal radius fractures.

Group	n	Patient satisfaction classification, n (%)			Total satisfaction rate (%)	Medical staff recognition classification, n (%)			Total staff recognition rate (%)
		Satisfied	Basically satisfied	Dissatisfied		Recognized	Basically recognized	Unrecognized	
Intervention group	56	45 (80.4)	10 (17.9)	1 (1.8)	98.2	50 (89.3)	6 (10.7)	0 (0.0)	100.0
Control group	56	40 (71.4)	12 (21.4)	4 (7.1)	92.9	46 (82.1)	9 (16.1)	1 (1.8)	98.2
χ^2^ value	–	–	–	–	6.045	–	–	–	6.045
*P* value	–	–	–	–	.014	–	–	–	.014

Satisfaction and recognition were scored using a 10-point scale; scores ≥8 were defined as “satisfied/recognized,” 6 to 7 as “basically satisfied/recognized,” and <6 as ‘dissatisfied/unrecognized’. The overall satisfaction rates and overall recognition rates between the 2 groups were compared using Pearson’s chi-square test. All *P* values are two-sided, and a *P* value < 0.05 was considered statistically significant. The χ^2^ and *P* values presented in the table correspond to the comparison of the total rates.

### 3.3. Comparison of TCM-related indicators between the 2 groups

After the intervention, the clinical delivery rate of TCM measures in the intervention group reached 100%, significantly higher than the 64% in the control group and the 65% in the intervention group before the intervention (*P* < .05). The TCM syndrome efficacy score also improved significantly in the intervention group (Table 7).

**Table 7:** Comparison of TCM nursing implementation rate and TCM syndrome efficacy score before and after intervention in elderly patients with distal radius fractures.

Group	n	TCM intervention implementation rate		TCM syndrome efficacy score	
		Pre-intervention	Post-intervention	Pre-intervention	Post-intervention
Intervention group	56	65.00%	100.0%*^,^†	63	95*^,^†
Control group	56	63.00%	64.00%	60	60
*P* value	–	.821	.04	.763	.04

Values are % or mean scores (range 0–100, higher = better). Chi-square test for rate; independent *t* test for score. Pre- and post-intervention: baseline and 8 weeks.

TCM = traditional Chinese medicine.

* *P* < .05 versus control group at the same time point.

† *P* < .05 versus pre-intervention within the same group.

### 3.4. Comparison of orthopedic-related indicators between the 2 groups

After the intervention, the VAS pain score and limb swelling score in the intervention group were significantly lower than those in the control group and their own pre-intervention levels. The Cooney wrist function score was significantly higher in the intervention group compared with the control group and its own pre-intervention level (*P* < .05; Table 8).

**Table 8:** Comparison of orthopedic functional indicators between the 2 groups.

Group	n	VAS pain score		Limb swelling score		Cooney wrist function score	
		Pre-intervention	Post-intervention	Pre-intervention	Post-intervention	Pre-intervention	Post-intervention
Intervention group	56	6.0 ± 1.2	2.0 ± 0.7*^,^†	3.0 ± 0.8	1.0 ± 0.4*^,^†	75.0 ± 8.5	95.0 ± 5.5*^,^†
Control group	56	7.0 ± 1.3	5.0 ± 1.1	4.0 ± 0.9	4.0 ± 0.9	73.0 ± 9.0	75.0 ± 8.0
*P* value	–	.062	**.030**	.057	**.030**	.071	**.030**

Values are mean ± SD. VAS: 0 to 10 (higher = worse pain); swelling: 1 to 4 (higher = severer); Cooney: 0 to 100 (higher = better function). Independent-samples *t* test for between‐group post-intervention comparisons. Bold *P* values indicate statistical significance (*P* < .05).

SD = standard deviation, VAS = Visual Analog Scale.

* *P* < .05 versus control group at post-intervention.

† >*P* < .05 versus pre-intervention within the same group.

### 3.5. Robustness analysis results: propensity score-matched concurrent control

After propensity score matching, 56 pairs of patients were successfully matched (intervention group: n = 56; concurrent control group: n = 56). Baseline characteristics were well balanced between the 2 matched groups, with all SMDs below 0.1 (Table 11).

**Table 11:** Comparison of outcomes between the intervention group and propensity score-matched concurrent control group.

Outcome	Intervention group (n = 56)	Concurrent control group (n = 56)	Effect estimate (95% CI)	*P* value
Total complication rate, n (%)	2 (3.6)	8 (14.3)	RR = 0.25 (0.07–0.89)	**.032**
TCM intervention delivery rate, n (%)	56 (100.0)	40 (71.4)	–	**<.001**
VAS pain score (mean ± SD)	1.8 ± 0.7	4.2 ± 1.1	MD = −2.4 (−2.8 to −2.0)	**<.001**
Swelling score (mean ± SD)	1.4 ± 0.5	2.6 ± 0.8	MD = −1.2 (−1.5 to −0.9)	**<.001**
Cooney wrist score (mean ± SD)	95.2 ± 6.8	82.1 ± 9.5	MD = 13.1 (10.1–16.1)	**<.001**
Patient satisfaction (satisfied, %)	96.4% (54/56)	78.6% (44/56)	RR = 1.23 (1.07–1.41)	**.005**

Visual Analog Scale (0–10, higher = worse pain); limb swelling score (1–4, higher = severer); Cooney wrist function score (0–100, higher = better function). Values for complication and implementation rates are presented as n (%) with RR; VAS, swelling, and Cooney scores are presented as mean ± SD with MD. *P* values were derived from the chi-square test for categorical variables and the independent-samples *t* test for continuous variables. Statistically significant *P* values (<.05) are shown in bold. The TCM intervention delivery rate in the intervention group reached 100%, precluding RR estimation; between‐group difference was tested by chi-square test.

CI = confidence interval, MD = mean difference, RR = relative risk, SD = standard deviation, TCM = traditional Chinese medicine, VAS = Visual Analog Scale.

Consistent with the primary analysis, the intervention group demonstrated significantly lower total complication rates compared with the matched concurrent control group (3.6% vs 14.3%, RR = 0.25, 95% CI: 0.07–0.89, *P* = .032). The TCM delivery rate remained significantly higher in the intervention group (100% vs 71.4%, *P* < .001). Improvements in VAS pain score (1.8 ± 0.7 vs 4.2 ± 1.1, *P* < .001), swelling score (1.4 ± 0.5 vs 2.6 ± 0.8, *P* < .001), and Cooney wrist function score (95.2 ± 6.8 vs 82.1 ± 9.5, *P* < .001) in the intervention group also remained statistically significant. Patient satisfaction with TCM treatment was higher in the intervention group (96.4% vs 78.6%, *P* = .005). These findings were directionally consistent with the primary historical control analysis, supporting the robustness of the main results against temporal confounding (Table 11).

## 4. Results of the QCC activity

### 4.1. Tangible outcomes

After the intervention, all outcome indicators reached or exceeded their target values. The 8 major objectives – reducing pain score, reducing swelling score, improving DRF healing rate, improving Cooney wrist function score, reducing DRF complication incidence, improving the clinical delivery rate of TCM, improving TCM syndrome efficacy score, and improving patient satisfaction with TCM treatment – were all achieved. Details are shown in Table 9.

### 4.2. Intangible outcomes

Comparing the comprehensive abilities of QCC members before and after the activity revealed improvements across various dimensions, as shown in Table 10 and Figure 3.

**Table 10:** Comparison of QCC team members’ comprehensive competency scores before and after activity.

Assessment dimension	Score before QCC activity	Score after QCC activity	Absolute improvement	Relative improvement rate (%)
Problem-solving ability	30	42	12	40.0
Responsibility awareness	38	46	8	21.1
Communication and coordination ability	42	50	8	19.1
Self-confidence	34	46	12	35.3
Team cohesion	30	46	16	53.3
Work enthusiasm	34	42	8	23.5
QC tool application proficiency	38	50	12	31.6
Team internal harmony	46	50	4	8.7

Scores range from 0 to 50 for each dimension, with higher scores indicating stronger competency. All post‐activity scores were significantly higher than pre‐activity scores (paired *t* test, all *P* < .05). Relative improvement rate = (absolute improvement/score before activity) × 100%.

QC = quality control, QCC = Quality Control Circle.

**Figure 3.**
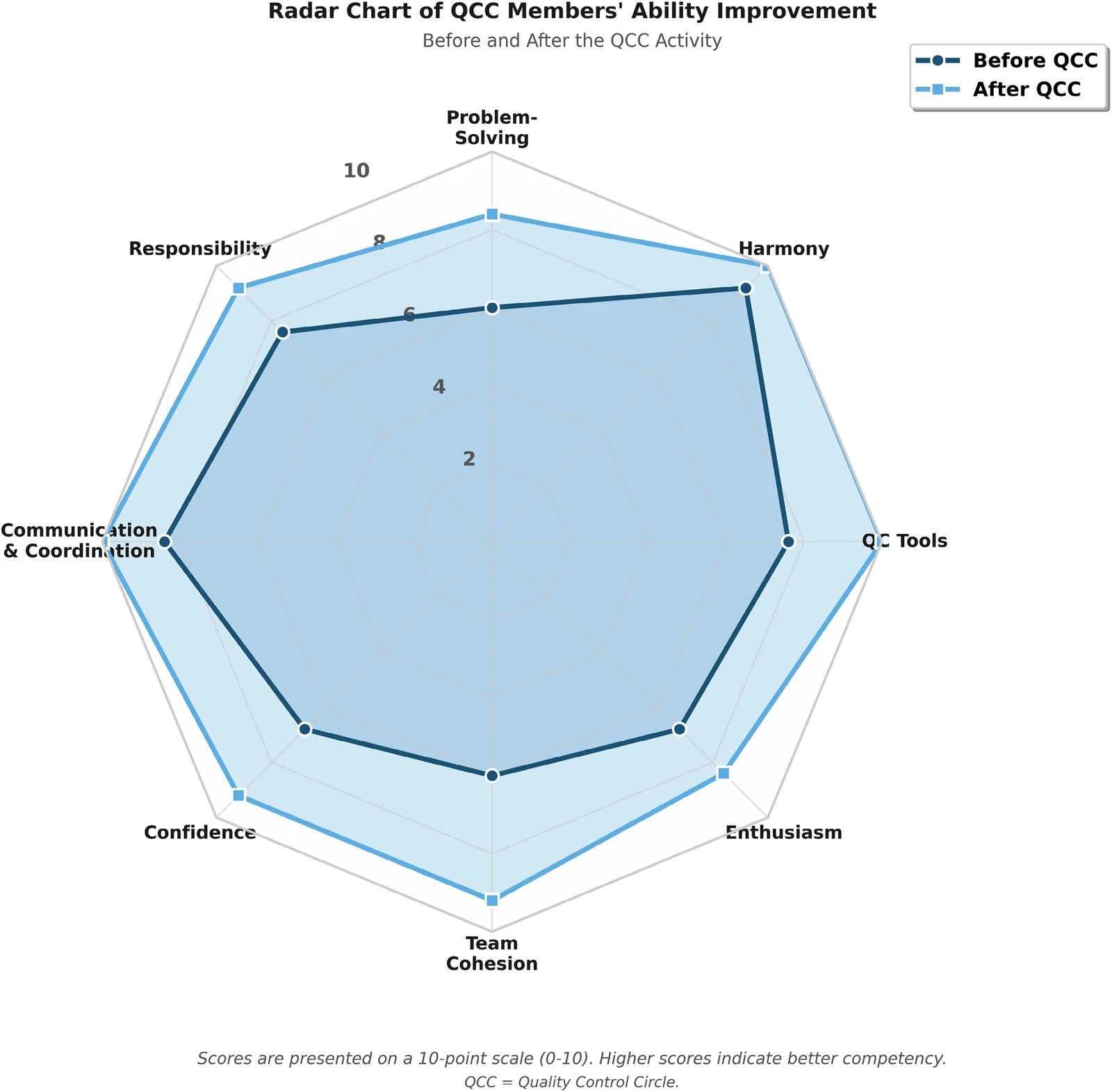
Radar chart showing the improvement in QCC members’ competencies across 7 dimensions before and after the QCC activity. The 7 dimensions include problem-solving ability, communication and coordination ability, professional knowledge, operational skills, team cohesion, innovation ability, and quality management awareness. The radar chart uses a 10-point scale (0–10) for competency assessment, with higher scores indicating better competency. QCC = Quality Control Circle.

### 4.3. Additional achievements

This QCC activity yielded fruitful additional results. In scientific research, it won first prize in the 2023 hospital QCC competition and second prize in the Jiangsu Provincial Hospital Association QCC competition, and led to the publication of 3 related academic papers. Regarding healthcare quality, as the TCM nursing quality control center in Changzhou, patient satisfaction with TCM treatment and nursing quality in our hospital significantly improved. In terms of influence, leveraging the hospital platform, the model attracted visits and exchanges from healthcare personnel both within and outside the province.^[15,16]^

The holistic principles underlying TCM nursing care have relevance beyond TCM-specific practice settings. Western healthcare systems increasingly recognize the value of comprehensive, patient-centered care models that address biological, psychological, and social dimensions of recovery. The QCC-driven framework described in this study demonstrates how traditional holistic care concepts – such as staged rehabilitation based on individual recovery patterns and the integration of external therapies with functional exercise – can be systematically implemented and quality-controlled within conventional healthcare structures. This structured approach to delivering multimodal care, with clear process documentation and outcome monitoring, may serve as a transferable model for interdisciplinary care coordination in other clinical contexts, even where TCM-specific interventions are not utilized. Future research should examine whether the quality improvement methodology itself, independent of specific TCM modalities, produces comparable benefits in non-TCM settings.

### 4.4. Standardized documentation

Through the QCC activity, 4 standardized operation manuals were developed and submitted to the hospital for review and approval: the Operation Procedure for Small Splint Fixation, the Operation Procedure for External Application of TCM, the Clinical Nursing Pathway for Elderly Distal Radius Fractures, and the Evaluation Standards for Specialist Nursing Quality.^[17]^

## 5. Discussion

The Healthy China Strategy emphasizes comprehensive and full-cycle health maintenance, aiming for a higher level of national health by 2030. In this study, the patient experience was significantly enhanced by measures such as installing self-service registration machines in wards and establishing one-stop TCM treatment and imaging services. Standardizing the reduction process ensured treatment efficacy. Integrating TCM cultural strategies and implementing TCM nursing measures such as external herbal applications effectively alleviated symptoms and promoted rehabilitation.^[18]^ This comprehensive TCM nursing model provides holistic, high-quality care and is worthy of clinical application. Furthermore, with the advent of the digital information age, optimizing medical data systems and utilizing digital platforms such as artificial intelligence greatly improve nursing efficiency, facilitating the implementation and promotion of this model.^[19]^ The emphasis on continuous care from admission to discharge is particularly crucial for the rehabilitation of elderly DRF patients.

The observed improvements in patient satisfaction and pain scores should be interpreted cautiously, as they may be partially attributable to the Hawthorne effect inherent in before-and-after study designs. Future studies employing blinded outcome assessment and concurrent control groups would help disentangle the specific effects of the intervention from nonspecific attention effects.

### 5.1. Limitations

Several limitations should be acknowledged. First, the non-randomized historical control design introduces potential temporal confounding. To address this concern, we performed a supplementary robustness analysis using propensity score matching to construct a concurrent control group from patients treated during the same period as the intervention group (March–December 2023). After matching, baseline characteristics were well balanced between the intervention and concurrent control groups (all SMDs < 0.1), and the results remained directionally and statistically consistent with the primary historical control analysis (Table 11). These findings support the robustness of our main conclusions against temporal confounding. Nevertheless, as with all observational studies, the possibility of residual confounding from unmeasured variables cannot be completely excluded. Future studies should employ multicenter prospective randomized designs with concurrent controls to further validate these findings.

Additionally, no subgroup or interaction analyses were performed due to the exploratory nature of this quality improvement study and the limited sample size within each subgroup; thus, whether the observed effects differ across age strata, fracture types, or comorbidity statuses remains unknown.

Second, certain outcomes (swelling, satisfaction) were assessed using non-validated departmental tools, posing measurement bias.

Third, the absence of long-term follow-up (>6 months) limits conclusions regarding sustained functional gain or late-onset complications such as post-traumatic arthritis.

Fourth, the single-center nature constrains generalizability.

Fifth, the Hawthorne effect is a well-recognized limitation in quality improvement research. Patients in the intervention group received more structured nursing contact, including case manager follow-up via WeChat, standardized health education videos, and digital symptom monitoring, compared with the control group, which received routine care. This differential attention may have independently influenced patient-reported outcomes (satisfaction, pain perception) and staff-reported outcomes (model recognition). We attempted to minimize this by ensuring both groups received identical orthopedic treatment protocols (Menghe School bone-setting and small splint fixation), so the core clinical intervention was not confounded by attention differences; using objective outcomes (complication rates confirmed by imaging, swelling scores assessed by 2 independent nurses) less susceptible to the Hawthorne effect; and implementing the QCC as a system-level process change rather than an individual-level intervention, which reduces but does not eliminate the influence of heightened awareness. Nevertheless, we cannot rule out that some observed improvements, particularly in patient satisfaction and self-reported pain, may partially reflect the Hawthorne effect rather than the specific intervention components.

Future studies should employ multicenter randomized designs with blinded outcome assessment, validated patient-reported outcome measures, concurrent controls or interrupted time series designs, and health economic evaluations.

Additionally, the observed effect sizes – particularly the 3.6% complication rate and the 20-point improvement in Cooney scores – should be interpreted with caution. The control group’s minimal functional improvement over 8 weeks suggests possible baseline imbalance in rehabilitation documentation, and the significant results for complication rates are dependent on a small number of events (n = 2 in the intervention group). Future randomized trials with blinded outcome assessors and larger sample sizes are needed to confirm the true magnitude of these effects.

## 6. Conclusion

In conclusion, this retrospective, single-center study describes the development and implementation of a comprehensive TCM nursing model for elderly DRF patients based on a project achievement-oriented QCC.^[11,16]^ The findings suggest that this model, which integrates characteristic TCM nursing techniques with modern quality management tools, is associated with improved short-term clinical outcomes, higher TCM nursing quality, and increased patient and staff satisfaction in this cohort. The model offers a systematic and standardized framework for practice. However, due to the methodological limitations outlined above – including the non-randomized historical control design, potential temporal confounding, Hawthorne effect, and lack of long-term follow-up – these results should be considered preliminary.^[20]^ The broader effectiveness, cost-effectiveness, and generalizability of this approach need to be confirmed through more robust study designs, including multicenter prospective randomized controlled trials with blinded outcome assessment, concurrent controls, longer follow-up periods, and formal health economic evaluations.

## Acknowledgments

The authors thank the Jiangsu Provincial Institute of Chinese Medicine Schools Open Research Project (Grant No. JSZYLP2024016), Jiangsu, China. All individuals named in the Acknowledgments have provided permission to be named.

## Author contributions

**Conceptualization:** Ya-Mei Bai.

**Data curation:** Na Cao, Ting Tang.

**Formal analysis:** Na Cao.

**Funding acquisition:** Ming Zhou.

**Methodology:** Fei-Yu Cheng.

**Project administration:** Ying Chen, Ming Zhou.

**Resources:** Ying Chen, Ming Zhou.

**Investigation:** Wen-Ting Wu, Fei-Yu Cheng.

**Validation:** Wen-Ting Wu.

**Visualization:** Wen-Ting Wu.

**Software:** Na Cao.

**Supervision:** Ya-Mei Bai, Yang Jiang, Han Sun.

**Writing – original draft:** Na Cao.

**Writing – review & editing:** Na Cao, Ya-Mei Bai, Yang Jiang, Han Sun.


