# Supplementary material for: Effect of a project achievement-oriented Quality Control Circle on traditional Chinese medicine nursing outcomes in elderly patients with distal radius fractures: A retrospective comparative study
Source: Medicine (Baltimore). 2026 Oct 2;105(40):e50905. doi: 10.1097/MD.0000000000050905 (PMC13632994; doi:10.1097/MD.0000000000050905)
Supplement: Supplementary file 1 [file medi-105-e50905-s001.docx]

**Appendix A: Glossary of Traditional Chinese Medicine Terms**

| TCM Term (Chinese) | English Translation | English Translation |
| --- | --- | --- |
| Menghe School (孟河学派) | Menghe School of TCM Orthopedics | A renowned TCM orthopedic school originating in Menghe Town, Changzhou, Jiangsu Province, China. Characterized by gentle manipulation techniques, minimal trauma, and holistic treatment approaches integrating bone-setting, herbal medicine, and functional rehabilitation. |
| Twelve bone-setting techniques (十二种正骨手法) | Twelve bone-setting manipulation techniques | The core manual reduction techniques of the Menghe School, including traction, rotation, compression, flexion, extension, and other maneuvers performed by trained orthopedists to achieve fracture reduction without surgical incision. |
| Small splint fixation (小夹板固定) | Small splint fixation | A traditional TCM method of fracture immobilization using bamboo or wooden splints padded with cotton cloth. Compared to plaster casts, it allows adjustable compression, better observation of skin condition, and earlier functional exercise. |
| Heiyu Ointment (黑玉膏) | Black Jade Ointment | One of Ma's Five-color Ointments; a black-colored TCM external plaster containing herbs such as Angelica sinensis (Danggui) and Carthamus tinctorius (Honghua) that promote blood circulation, reduce swelling, and relieve pain. Applied in the early stage (0–2 weeks) post-fracture. |
| Ma's Five-color Ointment (马氏五色膏) | Ma's Five-color Ointment | A series of five TCM external plasters (black, white, red, yellow, and green) developed by Dr. Ma, a renowned TCM orthopedist in Jiangsu Province. Each color corresponds to specific herbal formulations and indications for different stages of fracture healing. |
| Tendon Injury Bone-setting Oil (伤筋正骨油) | Tendon Injury Bone-setting Oil | A TCM liniment for external application containing herbs that soften tissues, relieve pain, and promote functional recovery. Used in the middle stage (2–4 weeks) of fracture healing, combined with guided functional exercises. |

Supplemental Digital Content 1. Appendix A: Glossary of Traditional Chinese Medicine Terms (PDF). This appendix provides English translations, pinyin, and brief explanations for TCM terms used in the manuscript, including Menghe School, twelve bone‑setting techniques, small splint fixation, Heiyu Ointment, Ma's Five‑color Ointment, and Tendon Injury Bone‑setting Oil.
